# De novo duplication of chromosome 16p in a female infant with signs of neonatal hemochromatosis

**DOI:** 10.1186/1755-8166-7-7

**Published:** 2014-01-23

**Authors:** Eva Maria Christina Schwaibold, Iris Bartels, Helmut Küster, Michael Lorenz, Peter Burfeind, Ronja Adam, Barbara Zoll

**Affiliations:** 1 Institute of Human Genetics, Georg August University, Heinrich-Düker-Weg 12, 37073 Göttingen, Germany; 2 Department of Pediatric Cardiology and Intensive Care Medicine, Georg August University, Robert-Koch-Str. 40, 37075 Göttingen, Germany; 3 Department of Pediatric Surgery, Georg August University, Robert-Koch-Str. 40, 37075 Göttingen, Germany; 4 Institute of Human Genetics, University of Bonn, Sigmund-Freud-Straße 25, 53127 Bonn, Germany

**Keywords:** 16p duplication, 16p dup, 16p whole arm duplication, Chromosome 16, Duplication of short arm of chromosome 16, Neonatal hemochromatosis, Iron overload, Neonatal liver failure

## Abstract

Reported cases of “pure” duplication of the entire short arm of chromosome 16 (16p) are rare, with only 7 patients described in the literature. We report on a female infant with de novo 16p duplication localized to the short arm of chromosome 6, detected by chromosomal analysis and characterized by array CGH and fluorescence in situ hybridization. This baby girl presented with clinical symptoms characteristic of patients with duplications of the short arm of chromosome 16: psychomotor retardation, constitutional growth delay and specific dysmorphic features, including proximally placed hypoplastic thumbs. In addition, she exhibited evidence of neonatal hemochromatosis as shown by direct hyperbilirubinemia, iron overload and elevated liver enzyme levels. To our knowledge, this is the first report of signs of neonatal hemochromatosis in a patient with 16p duplication.

## Background

Duplication of the short arm of chromosome 16 is a well described condition with severe prenatal and postnatal growth retardation, dolichocephalus, facial dysmorphisms, clinodactyly and hypoplastic thumbs [1]. Most of the cases are derived from a chromosomal translocation and present a concomitant deletion of another chromosome. To the best of our knowledge, only 7 of the reported patients have a “pure” duplication of the complete short arm of chromosome 16 [2-8]. These reported 16p duplications were all identified by means of chromosomal analysis and/or fluorescence in situ hybridization (FISH).

Neonatal hemochromatosis (NH) is a severe clinical and pathological entity that affects newborns and is characterized by liver failure associated with hepatic and especially extrahepatic iron deposition [9]. There is some overlap with adult hemochromatosis. Adult hemochromatosis is mostly due to gene mutations (e. g. in the *HFE* gene) [10]. In contrast, NH is considered to be primarily the result of a maternal-fetal alloimmune disorder - also termed gestational alloimmune liver disease (GALD) [9,11].

Here, we report the case of a female infant with neonatal liver failure suggestive of NH together with psychomotor retardation, external malformations and facial dysmorphisms. Cytogenetic analysis revealed the presence of additional chromosomal material at the terminal short arm of chromosome 6. Characterization by both aCGH and FISH analyses defined that additional chromosomal region as a *de novo* duplication of the entire short arm of chromosome 16.

## Case presentation

### Case report

The patient is the second child of healthy non-consanguineous parents. Her father was 46 years old and her mother 29 years old when she was born. She had one healthy older brother, and there was no family history of congenital anomalies, intellectual disability (ID) or miscarriages. Following an otherwise uneventful pregnancy, the mother had premature rupture of her membranes at the beginning of the 34^th^ week of gestation. A fetal ultrasound at that time demonstrated a female singleton fetus with intrauterine growth retardation and anomalies of the central nervous system, heart and midface. A caesarean section was performed at the end of the 34^th^ gestational week due to pathologic cardiotocograph changes.

At birth, the baby weighed 1860 g (10-25th percentile) and measured 39 cm in length (<3rd percentile), and her head circumference measured 30.5 cm (25-50th percentile). Her Apgar scores were 5/7/9, and her cord blood pH was 7.35. The newborn screening was normal. Due to respiratory problems, she was transferred to the neonatal intensive care unit. As the respiratory situation did not improve and weaning from CPAP-support was not possible a tracheostomy was performed.

**Figure 1:**
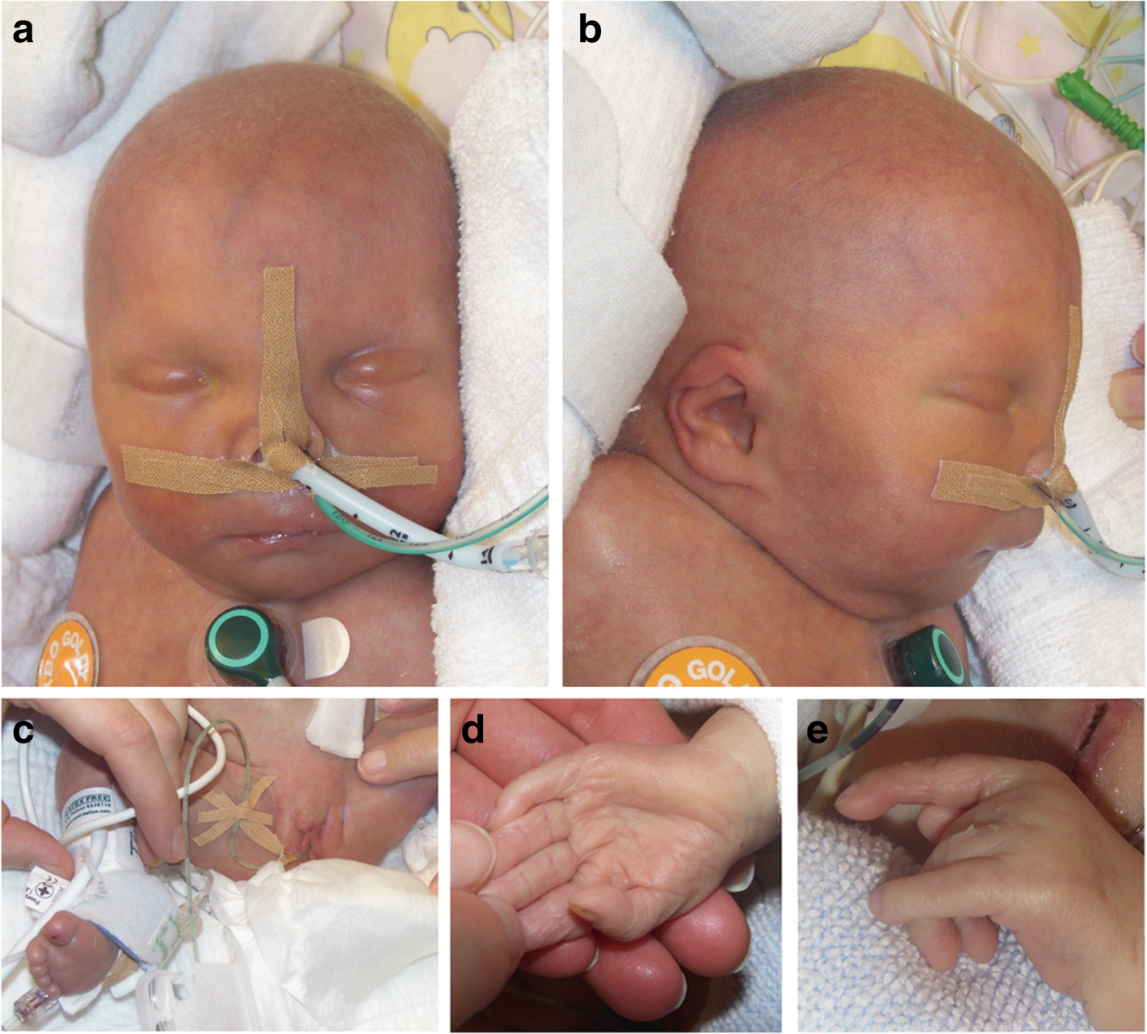
**Representative photographs of the patient at 3 days of age (a-c) and 2 months of age (d-e), respectively. (a-b)** The main facial features of the 3-day-old female newborn were narrow palpebral fissures, microretrognathia, hypertelorism with slight upslanting of her palpebral fissures, low-set, dysplastic ears with folded upper helices, a round face and a small nose with an almost absent nasal bridge. **(c)** The external genitalia were dysplastic. A sandal gap was present between the first and second toe. **(d)** The thumbs were hypoplastic and proximally placed. **(e)** The second and fifth fingers were outstretched and deviated towards the middle fingers; a clinodactyly of the distal phalanges could be observed.

At three days of age, the patient’s facial appearance (Figure 1a-b) was characterized by narrow palpebral fissures, hypertelorism with slight upslanting of her palpebral fissures (Figure 1a), microretrognathia (Figure 1a-b), dysplastic, low-set ears with folded upper helices (Figure 1b), a small nose and a round face (Figure 1a-b). Her neck was short with an excess of skin (Figure 1a-b). She had a median cleft of both the soft and hard palates. Her outer genitals were ambiguous with dysplastic labia (Figure 1c), and she had a mild sacral meningocele. Severe abnormalities of both hands could be observed, including proximally placed hypoplastic thumbs (Figure 1d) and a deviation of her second and fifth fingers towards the middle fingers (Figure 1e). Her second and fourth fingers were always outstretched, and her distal phalanges showed clinodactyly (Figure 1d-e). Sandal gaps were noted between her first and second toes (Figure 1c).

Postnatal echocardiography demonstrated a complex cardiac defect with ectasia of the root of the pulmonary artery, a dysplasic aortic valve, a persistent left vena cava, an atrial and subaortic ventricular septal defect. A persistent Ductus arteriosus (PDA) required surgical ligation.

Cranial ultrasonography revealed agenesis of the corpus callosum, enlargement of the posterior ventricles, a large cisterna magna and sparse gyration. Her electroencephalogram was normal. Due to the presence of anal atresia with a subvaginal fistula, the baby required a colostomy on her third day of life. Ultrasound of the abdomen was normal.

Direct hyperbilirubinemia was found, and it lasted for three months. Serum transaminases, lactate dehydrogenase and γ-glutamyl transferase also showed prolonged elevation. Beginning at 4 weeks of life, her ferritin levels were markedly elevated and fluctuated between 1984 and 4254 μg/L (reference range: 10–204 μg/L). Her transferrin saturation peaked at 92% (reference range: 11–52%) at 7 weeks of life. Laboratory tests for toxoplasmosis, syphilis, coxsackie virus and echo virus showed normal results.

Unexpectedly, at 5 months of age, her physical condition deteriorated, and she died within a few hours from cardiovascular failure.

### Methods

Blood samples were collected from the patient and her parents after obtaining the parents’ signed informed consent. The manuscript is a retrospective case report that does not require ethics committee approval at the University of Göttingen. For rapid karyotyping in the newborn, FISH analysis was performed using multicolor DNA probes for chromosomes 13, 18, 21, X and Y (AneuVysion Vysis/Abbott, Wiesbaden, Germany). Metaphase chromosome spreads were prepared from phytohemagglutinin (PHA)-stimulated peripheral blood cultures using standard protocols, and 10 GTG-banded metaphases were analyzed.

Genome-wide copy number scans were performed with the patient’s and her parents’ lymphocyte DNA using an Agilent SurePrint G3 Human CGH Microarray Kit 4×180K and was read using an Agilent Microarray Scanner G256BA along with Agilent Feature Extraction Software V9.1 (Agilent Technologies, Inc., Santa Clara, CA) according to the manufacturer’s instructions. The results were analyzed using Agilent Cytogenomics 2.0 software. To confirm the aCGH results of the newborn FISH was performed using the DNA probes 16pter and 22q11.2 (BCR as a control; Abbott Molecular, Wiesbaden, Germany; Q-Biogene, Amsterdam, The Netherlands).

### Results

**Figure 2:**
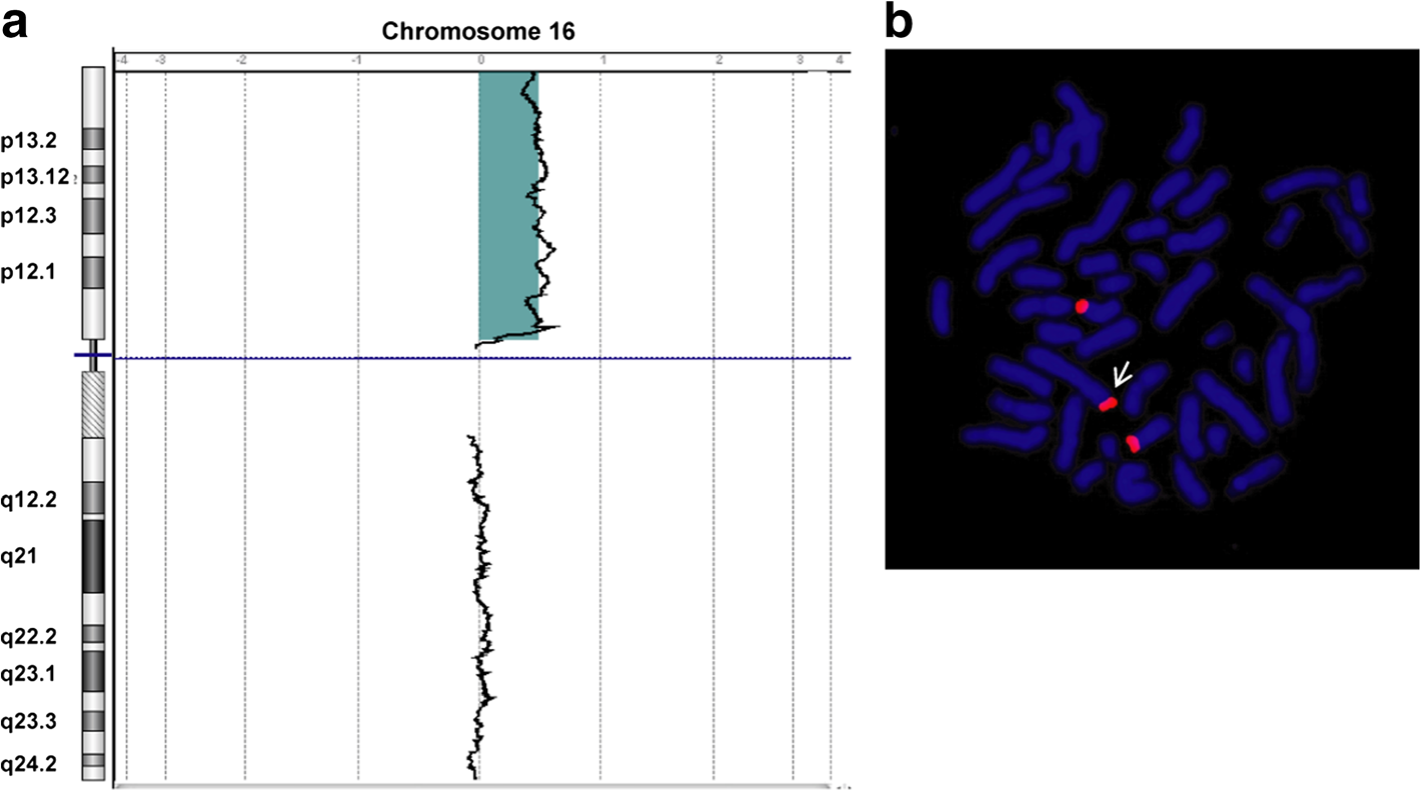
**aCGH and FISH analysis of chromosome 16 in our patient. (a)** aCGH identified a duplication of chromosome region 16pter-p11.2 Log 2 ratio data for two dye-swap plots (patient/control) are presented according to their positions in the human genome. The black line indicates the moving average. The greenish shaded region indicates the duplication on chromosome region 16pter-p11.2. The blue horizontal line marks the location of the centromere. **(b)** Representative image of chromosome metaphase demonstrating hybridization of the subtelomeric probe 16pter (red) in the FISH analysis. The white arrow indicates the location of the additional chromosomal region 16pter-p11.2 translocated onto the telomeric region of chromosome 6p.

FISH analysis of the newborn’s interphase chromosome spreads was performed immediately after birth with probes for chromosome 13, 18, 21, X and Y but yielded normal results. GTG-banded metaphases prepared from lymphocyte cultures demonstrated distal elongation of the short arm of chromosome 6, preliminary karyotype 46,XX,add(6)(p25). Because cytogenetic analysis alone could not identify the origin of this chromosomal region, aCGH was performed, which demonstrated duplication of the entire short arm of chromosome 16 (16pter to 16p11.2) with a size of approximately 33.83 Mb (Figure 2a) involving positions 119.664 to 33.923.140. No additional chromosomal aberration except the duplication of chromosome 16p could be detected. Cytogenetic analyses and aCGH of the parents’ chromosomes did not show any pathogenic chromosomal aberrations (data not shown).

To confirm these results, FISH analysis using a subtelomeric probe for chromosome 16p was performed on metaphase spreads of the patient’s lymphocytes (Figure 2b). In addition to the two normal hybridization signals detected on chromosomes 16p, an extra hybridization signal was detected on the p-telomere of the structurally abnormal chromosome 6 (Figure 2b). The final interpretation of the karyotype was 46,XX,add(6)(p25)dn.ish dup(16)(p13)(16pter+).arr[19] 16pterp11.2(106,320×2,199,664-33,923,140×3,33,939,146×2) (ISCN2013).

## Discussion and conclusions

Reported cases of complete, “pure” duplications of the short arm of chromosome 16 are very rare, with only 7 patients described in the literature [2-8]. With the exception of the patients of Dallapiccola et al. [4] and Llamas et al. [5], the duplication of 16p in all of these patients was due to a maternal translocation. The duplication of 16p in our patient was characterized by FISH analysis and aCGH (Figure 2), methods that were not available at the time of Dallapiccola et al.’s [4] and Llamas et al.’s [5] studies. Many of the clinical features described in these patients were found to be present in our patient (Table 1). In particular, the characteristic proximally placed hypoplastic thumbs were observed in our patient (Figure 1d). The facial features of our patient (Figure 1a-b) were similar to those of the patients in those case reports. Although heart defects have been described previously in patients with 16p duplications (e.g., [6]), the heart defect in our patient was more severe.

**Table 1:** Main clinical findings of the 8 reported patients with complete trisomy 16p

	**Magnelli, **[2]	**Yunis et al., **[3]	**Dallapiccola et al., **[4]	**Llamas et al., **[5]*	**Jalal et al., **[6]	**Léonard et al., **[7]	**Rochat et al., **[8]	**Our patient**
Inheritance	Maternal b. t.	Maternal b. t.	De novo	De novo	Maternal b. t.	Maternal b. t.	Maternal b. t.	De novo
Gender	Male	Female	Female	Male	Female	Female	Male	Female
Living/deceased	Alive at 7 years	Alive at 5 months	Alive at 21 months	Alive at 6 months	Alive at 7 months	Died at 3 days	Alive at 26 years	Died at 5 months
Postnatal growth restriction	+	+	+		-	+	+	+
Facial dysmorphisms:								
Round face		+	+	+	+	+	+	+
Short neck	+		+	+	-		-	+
Low set/dysmorphic ears	+	+	+	+	+	+	+	+
Micro-/retrognathia					+	+	+	+
Hypertelorism		+	+	+	+	+	+	+
Depressed nasal bridge		+	+	+	+		+	+
Anteverted nostrils		+	+	+	+		+	
Narrow palpebral fissures		+		+	+		+	+
Upslanting palpepral fissures			-		+	+	+	+
Cleft palate	+				+	+	+	+
Excess of skin						+	+	+
Neurologic defects:								
Structural CNS anomalies	-					+		+
Psychomotoric retardation/intellectual disability	+	+	+	+	+		+	+
Hand anomalies:								
Proximally placed/hypoplastic/absent thumb	-	+	-			+	+	+
Overlapping fingers					+	-	+	+
Heart defect					+	-	-	+
Respiratory problems						+		+
Abdominal anomalies	+	+	+			+	+	-
Genitourinary malformations	+			+		+	+	+
Orthopedic anomalies of the lower extremities	+	+	+	+	-	+	+	-
(Symptoms of) neonatal hemochromatosis			-					+

+ denotes present, - absent, empty field = not reported; b. t. = balanced translocation; CNS = central nervous system; * from [6]; table modified from Rochat et al. [8].

Additionally, our patient exhibited evidence of iron overload and neonatal liver failure. These pathological findings could have several reasons, e.g. NH as being one of the most common causes of liver failure in newborns [12]. For a definitive diagnosis of NH as the cause of neonatal iron overload histological confirmation of extrahepatic iron accumulation is crucial [13]. This could have been examined by a salvatory gland biopsy [13]. A T2-weighed magnetic resonance imaging of the abdomen showing organs with siderosis with lower signal intensity than the spleen or the analysis of the alpha-fetoprotein level (normally markedly elevated in NH patients) would also have been helpful to verify the diagnosis of NH [13]. Unfortunately, neither of these examinations was performed in our patient. Therefore, it has to remain unclear whether our patient really had NH or only displayed evidence of neonatal liver failure resulting from a different etiology. At least we can rule out some metabolic and infectious causes of neonatal liver failure [12] by a normal newborn screening and normal laboratory results for toxoplasmosis, syphilis, coxsackie virus and echo virus, respectively.

To our knowledge, NH or symptoms suggestive of NH has/have never been reported in patients with duplication or even a partial duplication of chromosomal arm 16p. NH is most commonly due to maternal alloantibodies against the affected fetus [9], but it has also been described in Down syndrome patients with megakaryocytic transient myeloproliferative disorder [14,15]. Miyauchi and coworkers [15] hypothesized that an immunological deficit, a liver fibrosis caused by megakaryocytes or a gene-dosage effect may be responsible for the symptoms of NH in their patients. These researchers concluded that patients with Down syndrome represent only a subset of patients with symptoms of NH among NH patients of many different etiologies.

With regard to genetic counseling of the parents of the proband, it would have been important to clarify the cause of the neonatal liver failure in our patient. The high risk of recurrence (up to approximately 80%) of NH and the availability of prophylaxis of GALD-related NH by prenatal treatment with intravenous immunoglobulin [9,11] make that question even more pressing. A histopathological examination of the patient’s liver and extrahepatic tissues for evidence of iron overload and activation of the fetal complement cascade in response to maternal alloantibodies might have been informative. Unfortunately, the parents refused to allow an autopsy to be performed on their child. The target protein of GALD-associated NH is still unknown, and prenatal screening for maternal fetal alloantibodies is not yet available.

Consequently, a definite recommendation regarding maternal prenatal treatment with intravenous immunoglobulin during future pregnancy could not be made, and the mother’s prognosis for the recurrence of a neonatal liver failure in a subsequent offspring could not be established. The risk of recurrence of 16p duplication in future pregnancy is very low because this chromosomal aberration has occurred de novo. However, a low probability of germ cell mosaicism in one of the parents remains.

## Consent

Written informed consent was obtained from the parents of the patient for publication of this Case report and any accompanying images. A copy of the written consent is available for review by the Editor-in-Chief of this journal.

## Competing interests

The authors declare that they have no competing interests.

## Authors’ contributions

EMCS evaluated the aCGH analysis, participated in the genetic counseling of the patient’s parents and drafted the manuscript. IB was responsible for the cytogenetic analysis and helped with the final version of the manuscript. HK helped with the final version of the manuscript. HK and ML were responsible for the medical treatment of the patient. PB was responsible for the aCGH analysis and helped with the final version of the manuscript. RA participated in the evaluation of the aCGH analysis. BZ did the genetic counseling of the patient’s parents. All authors read and approved the final manuscript.
